# The Theory of Reproduction

**Published:** 1878-11

**Authors:** S. Z. Ammen


### THE THEORY OF REPRODUCTION.

By S. Z. AMMEN.

Discoveries in biology within the last fifty years have relieved the processes of reproduction and gestation of some of the mystery which formerly surrounded them. Reproduction of the human species is shown to be essentially but a partition of the parent organism; gestation, the retention of the detached fragment in a part capable of supplying organic matter in an easily assimilable form. In this view, the relation of offspring to parent is not that of a product to the producer, or of a thing created to a creator, but that of a part to the whole. The germinal cell, or spermatozoon, is to be considered as much identical in substance with the parent, as are the small crystals of copper sulphate with the large one from the solution and recrystalization of which they are resulted.

In an animal of highly complex structure like man, the correctness of this view is not so evident as at first sight. There have been developed in him so many highly specialized structures, for other purposes, that the nature of the act of reproduction is apt to be misunderstood from its very simplicity. By the study of organisms of little or no specialization of structure, biologists have been able to simplify their problem, and attain positive results. Indeed, it has been found that the essential material of life, bioplasm, or protoplasm, which gives all its vigor to the cell, may and does exist without any specialization of structure whatever. It has been found in vast quantities at the bottom of the Atlantic as a kind of formless but living slime, capable of assimilating materials for its growth from the sea-water.

Here we have reproduction in its simplest manifestations: a mass of organic matter in contact with inorganic matter raises the latter to the organic state. We are concerned at present with the curious question of the nature of "vitality." It is sufficient to call the attention to this Urschleim of the Germans, this cell-material not yet formed into cells, which possesses the vital property of a capability of assimilation. "Chemical action is wasting its substance and dissipating its energy on the one side, and rebuilding and reconstructing its parts on the other." Its material particles are continually being wasted and excreted, while new particles are as incessantly being added to its mass. It is a living substance without any determinate form. A small mass of it subjected to new chemical conditions, may readily be supposed to have formed about its exterior a thin pellicle, which will protect the protoplasm within from change. If the new conditions become permanent (conditions occasioned by new currents of water, with matter of slightly different chemical constitution in solution), we may expect all protoplasm not thus protected by exterior (cell) walls to be destroyed. A cell thus formed would assimilate and excrete the material of its growth through its cell-wall, by the familiar processes of diffusion, endosmosis and exosmosis. So much for its nutrition. Its growth, however, would seem to be prevented by the narrow limits of its wall. The solution of this difficulty may be very prettily illustrated by a chemical experiment. Introduce carefully a large drop of an aqueous solution of tannic acid within an aqueous solution of gelatine. On coming in contact, the acid and gelatine will form a leathery coating around the drop, completely inclosing it, and separating, of course, the acid within from the gelatine without. In short, an artificial cell is formed. If we watch the behavior of this cell, we shall first see that the gelatine, without passing within through the cell-wall, by a process of endosmose causes a thickening of the cell-wall and a compression of its contents. This is presently relieved by a bursting of the cell-wall, so that a drop of the acid protrudes, like a bud. Around this, in turn, a new wall is formed, and the process of budding will go on till the supply of acid within is exhausted. In like manner it is, we may suppose, that cell-life proceeds. There is this important difference, however, in favor of the cell containing protoplasm over that containing tannic acid, that the former can manufacture materials for its growth from its surroundings indefinitely, while the latter must stop when its little original supply is exhausted. But the comparison holds far enough to illustrate the process by which the reproduction of cells takes place. The new cell buds out from the old, receiving part of its substance. In many of the lower plants and animals reproduction takes place by fission; the parent mass simply splits in two, and each half is entitled to the name of parent, as well as son. Many organisms (com posed of more than one cell) multiply by budding. Thus the zoophyte of our coast develops numerous buds on his exterior. These after awhile fall off and float away to grow up in the sea—the analogue, for such embryos, of the womb in the human species. The zoophyte, it will be observed, reproduces by buds upon his (or her) exterior, and hence performs no function of gestation. Other animals develop buds upon their interior surface, and supply them with material for growth in the shape of easily assimilable organic matter. To this class man belongs. Yet another class, budding interiorly, resolve themselves wholly into offspring, the integument alone surviving. Here the weight of the offspring is almost equal to that of the parent. In these last two classes there is a veritable gestation, a provision of conditions specially favorable to nutrition. All the higher animals demand thus, during the first period of their growth, a more plentiful supply of assimilable matter than is to be found diffused in the water of the sea, or in river water, or in the soil. Animals nourished only in these various wombs of mother earth are puny in size and simple in structure. The proper growth of a cell demands that it be plentifully supplied with nourishment from the moment of its detachment from the parent mass.

The facts here cited are sufficient to enforce the truth that the so-called young of any organism is not essentially a different individual from its so-called parent, but is a part, a fraction, of the parent. We give arbitrarily the name of parent to the larger fraction, which is commonly, also, the more highly specialized in structure and function. The detached part (in man) is a cell filled with organic matter in its least specialized form—protoplasm. The parent mass, however, besides a remainder of such undifferentiated cells, is largely made up of cells so highly specialized in function as to have lost their power of reproduction—a function belonging originally to all cells. A cell of the arm, for instance, if placed in the womb, would not develop into an infant, being incapable of assimilating the nutriment as there provided. But it does assimilate from the blood, and reproduces other cells like itself; *i. e.*, it retains its reproductive powers, but in a special line only. Among cells as among men, a special training disqualifies for any large variety of actions. Only in the testicle and ovary is the human protoplasm found in an unspecialized (and hence fully developable) form. The cells of the rest of the body, being devoted to special growths, are no longer equal to the general function of reproducing the entire man. This is limited to the cells thrown off from time to time from the male and female generative organs. Theoretically, cells from the organs of either sex should be developable, and there are many indications that the human race was formerly hermorphraditic, as we know many animals and most plants now are. Herbert Spencer suggests an explanation of the necessity, at present, of commingling the contents of spermatic cells of two kinds—those of the ovum with those of the spermatozoon—in order to full dovelopment.

From what has been said in the foregoing pages, it is evident that so far as the parent is concerned, the act of reproduction ceases with the detachment of a developing cell from the parent mass. What follows is a story of nutrition. The cell has now a separate life.

There are many modes of nutrition :

1. There is no gestation. The detached cell is dismissed by the parent at the period of "ovulation" into the outer world, to find there as best it may the necessary conditions of warmth, moisture and nutriment.

II. There is partial gestation. The cell is dismissed surrounded by a store of nutriment, as in the eggs of birds.

III. Gestation is more or less complete. The cell is retained within the body of the parent, and there supplied with the condition of growth, till it is partially developed and can provide more or less perfectly for its wants.

There is an ascending series in which thc apparatus for supplying the conditions of development, in connection with the parent, is rendered more and more complete, till man and the kangaroo are reached. The provision of a marsupium, or bag, in which the young are cared for after birth, seems to raise the last-mentioned animal, anatomically at least, above the *genus homo*, and all other similar mammalia.

In the human male there is no provision for the life of the detached cell. In the female there is; and it is on this provision that the difference of the sexes turns.

In the female, a part of the tube along which the cell is to pass to be voided, is enlarged, so as to form a vessel capable of containing it till a late period of its development. This vessel, the womb, supplies the necessary conditions of warmth and moisture and a nutritive encompassing fluid, from which the cell may absorb and assimilate all that is needed for its growth.

